# Fetal Hemoglobin Modulation in Sickle Cell Disease: βs Haplotypes, Key Polymorphisms Identified by GWAS, and Advances in γ-Globin Editing: An Updated Overview

**DOI:** 10.3390/genes17020135

**Published:** 2026-01-27

**Authors:** Yusselfy Márquez-Benitez, Valeria Isabela Osorio-Garzón, Jaime Eduardo Bernal-Villegas, Ignacio Briceño-Balcázar

**Affiliations:** 1 Doctoral Program in Biosciences, Faculty of Engineering, Universidad de La Sabana, Campus del Puente del Común, Km 7 Autopista Norte de Bogotá, Chía 250001, Cundinamarca, Colombia; 2 Human Genetics Research Group, Faculty of Medicine, Universidad de La Sabana, Campus del Puente del Común, Km 7 Autopista Norte de Bogotá, Chía 250001, Cundinamarca, Colombia; valeriaosga@unisabana.edu.co; 3 Faculty of Health Sciences, Universidad del Sinú Elías Bechara Zainum, Transversal 54 #41-117, Cartagena 130001, Bolívar, Colombia

**Keywords:** sickle cell disease, haplotypes, polymorphisms, fetal hemoglobin, gamma-globins, gene editing, genome-wide association study

## Abstract

Fetal hemoglobin (HbF) plays a central role in mitigating the pathophysiological effects of sickle cell disease (SCD). Understanding the genetic determinants influencing HbF expression is essential for identifying the factors contributing to its modulation. This review provides an updated synthesis of evidence on HbF modulation, focusing on β^s^ haplotypes and their molecular characterization through Sanger sequencing, polymorphisms consistently associated with HbF levels in genome-wide association studies (GWAS), and recent advances in gene editing targeting HbF expression. An integrative review (2016–2025) was conducted using PubMed/MEDLINE, Scopus, and Web of Science, encompassing original research, experimental studies, systematic reviews, and genomic analyses. Key regulatory loci such as BCL11A, HBS1L-MYB (HMIP), and the HBB cluster explain a significant proportion of HbF variability across populations. Furthermore, additional variants in KLF1, NFIX, BACH2, and ZBTB7A have emerged as potential modulators in specific cohorts. Regarding advances in γ-globin editing, “prime editing”, although still in the experimental phase, has recently emerged as an innovative approach capable of introducing multiple HPFH-like mutations within γ-globin promoters, expanding future therapeutic possibilities in SCD. This review also provides a comparative overview of prime editing and other gene-editing strategies for HbF modulation, such as CRISPR-Cas9 and Base editing. Collectively, this work outlines the current landscape of HbF modulation and provides an informative basis for future research aimed at advancing precision-oriented therapeutic strategies in sickle cell disease.

## 1. Introduction

Sickle cell disease (SCD), also known as sickle cell anemia or drepanocytosis, is the most prevalent congenital hemolytic anemia worldwide. It is estimated that approximately 7 million individuals are affected [1].

Due to its substantial burden, the World Health Organization (WHO) has designated SCD as a major global public health concern [1,2].

SCD is a chronic disorder caused by a point mutation in the β-globin gene (HBB: c.20 A > T, rs334, 11p15.4) [2], which disrupts hemoglobin synthesis and leads to progressive, irreversible damage across multiple organ systems. The disease compromises physical, mental, and emotional health, and can be life-threatening [3]. Clinical complications include acute vaso-occlusive syndromes, increased susceptibility to infections, multisystem injury from ischemia, tissue infarction [1,2], cholelithiasis (and more uncommonly hepatolithiasis) that leads to cholecystectomy [4], and hemolysis that requires repeated blood transfusions, which can cause alloimmunization and increase the risk of post-transfusion infections, such as hepatitis [5] and chronic liver disease due to iron overload [6].

A central challenge in SCD management is its marked clinical heterogeneity, which influences the severity of vaso-occlusive complications, the extent of organ damage, and overall survival. This variability is strongly associated with molecular determinants that modulate fetal hemoglobin (HbF) levels. Elevated HbF concentrations are consistently linked to milder phenotypes, reduced hemolysis, and improved long-term outcomes, whereas lower HbF levels contribute to more severe disease expression [1,2].

The recognition of HbF as a key modifier has spurred decades of research aimed at identifying and targeting the genetic determinants that influence its modulation [1,2,3,7]. Early studies of beta-globin cluster haplotypes used techniques such as restriction fragment length polymorphism (RFLP) [3] and Sanger sequencing [7]. This research subsequently expanded to the recognition of key polymorphisms associated with γ-globin gene expression regulation through genome-wide association studies (GWAS) [8].

The molecular basis for gamma globin gene activation and the interaction of these and other genes have driven the development of innovative therapeutic strategies focused on reactivating HbF production, including targeted gene editing using advanced technologies such as CRISPR-Cas9, base editing, and, more recently, prime editing, which represent a promising advance in the activation of fetal hemoglobin as a treatment for SCD [9].

Based on this rationale, the objective of the present review is to provide an updated overview of HbF modulation, including the molecular characterization of βs haplotypes and their current identification methods (Sanger sequencing), HbF-associated polymorphisms described in genome-wide association studies (GWAS), and the most recent advances in γ-globin promoter editing (prime editing) in comparison with CRISPR-Cas9 and base editing.

This synthesis aims to integrate current knowledge, deepen understanding of the genetic basis of clinical heterogeneity, identify emerging therapeutic targets, and support the development of personalized strategies to improve prognosis and quality of life for patients with SCD.

## 2. Materials and Methods

This narrative review aimed to provide an updated overview of HbF genetic modulation in sickle cell disease.

Three important aspects were considered in the formulation of the article for inclusion within the genetic modulators of HbF in sickle cell anemia: beta globin haplotypes, polymorphisms in whole genome association studies, and gene therapy.

To achieve this objective, a search was conducted in databases such as Scopus, PubMed, and Google Scholar, taking into account MeSH terms and their association with “sickle cell disease”, “clinical variability”, “polymorphisms”, “fetal hemoglobin”, “gene therapy”, “beta-globin haplotypes”, “sequencing”, and “whole genome.”

The inclusion criteria were original research studies, systematic reviews and meta-analyses, narrative reviews, and scoping reviews between 2016 and 2025 with the following objectives:

(1) Beta globin haplotypes and their impact on fetal hemoglobin levels.
(2) Association of different polymorphisms observed in whole genome studies and their influence on gamma globin gene expression.
(3) Gene therapy related to increasing fetal hemoglobin levels in patients with sickle cell anemia.

Case reports, research in animal models, and articles with unclear methodology were considered as exclusion criteria.

The selected articles were first evaluated by title and abstract, then evaluated in full text according to inclusion and exclusion criteria.

A total of 60 articles were obtained, which allowed us to meet the initial objective of organizing the synthesized and updated information into three major topics related to the genetic modulation of fetal hemoglobin:

(a) Molecular description of classical β-globin haplotypes and their impact on HbF.
(b) Polymorphisms associated with fetal hemoglobin identified by GWAS.
(c) Gene-editing strategy for reactivating fetal hemoglobin.

## 3. Fetal Hemoglobin and Its Clinical Impact in SCD

Fetal hemoglobin (HbF) is produced by the γ-globin genes, HBG1 and HBG2, located within the β-globin gene cluster on chromosome 11 (11p15.4) [10]. Physiologically, HbF levels decline during the first months of life as they are gradually replaced by adult hemoglobin [10].

In SCD, pharmacological and genetic methods aim to increase HbF levels, as this hemoglobin has a higher affinity for oxygen and, unlike sickle hemoglobin (HbS), does not polymerize under hypoxic conditions. Thus, HbF preserves red blood cell shape and flexibility, reducing vaso-occlusion and hemolysis, mitigating the cascade of tissue damage characteristic of the disease [9,10].

Patients with higher HbF levels consistently experience milder disease manifestations; in contrast, those with very low HbF tend to present with more aggressive phenotypes, marked by recurrent vaso-occlusive episodes and progressive organ injury [11]. This strong correlation between HbF levels and disease expression underscores its role as the most powerful natural modifier of SCD severity.

## 4. Genetic Modulators of Fetal Hemoglobin

### 4.1. Classical β-Globin Haplotypes

The combination of multiple polymorphisms across the β-globin cluster gives rise to haplotypes that are inherited in an autosomal fashion, adjacent to the βs mutation. These were initially identified using specific restriction enzymes [12].

Several classical haplotypes, as well as minor ones, have been described in association with the βS allele. They show a strong geographic distribution in Africa and Asia and are designated as follows: Benin (BEN), Central African Republic or Bantu (BAN), Senegal (SEN), Cameroon (CAM), Arab-Saudi (Arab), Asian-Indian (AI), and atypical haplotypes (ATP) described in more recent studies [3,13].

Clinical severity varies among these haplotypes: the Bantu haplotype is generally associated with severe symptoms, Benin and Cameroon with intermediate severity, and Senegal and Arab-Indian with milder phenotypes and reduced organ damage [7,14].

#### 4.1.1. Cis-Regulatory Polymorphisms

Several polymorphic sites located in introns or promoters of the γ or β globin genes have been shown to affect the Gγ:Aγ chain ratio or the timing of the fetal-to-adult hemoglobin switch. However, among these, the XmnI polymorphism (rs7482144), detected by restriction fragment length polymorphism (RFLP) analysis, is the only variant with a clearly demonstrated functional effect [15].

Additionally, the haplotypes create an allele context (linked SNPs) across the β-globin cluster, where different combinations of these variants can affect the efficacy of the fetal gene silencing after birth [16].

#### 4.1.2. Molecular Identification of Classical β-Globin Haplotypes by DNA Sequencing

Traditional molecular methods to identify β-globin haplotypes primarily relied on restriction fragment length polymorphism (RFLP) analysis [17]. However, since 2017, DNA sequencing has increasingly been employed for identification based on single-nucleotide polymorphism (SNP) analysis [18]. The specific SNPs used for this analysis are summarized in Table 1.

The application of next-generation sequencing (NGS) technologies has also been incorporated into the study of SCD, enabling the simultaneous analysis of multiple genes, loci, and the whole genome [19,20]. For haplotype identification, NGS studies have focused on analyzing the polymorphisms described in Table 1, utilizing sequencing by Synthesis (SBS) [21].

Table 2 summarizes the general characteristics of the sequencing technologies recently used in haplotype identification in SCD.

#### 4.1.3. Classical β-Globin Haplotypes and Fetal Hemoglobin

The influence of β-globin haplotypes on the clinical severity of SCD is largely attributed to their effect on HbF levels [22].

Specifically, the Arab-Indian (AI) and Senegal (SEN) haplotypes are generally associated with fewer clinical complications compared to other haplotypes. This protective effect has been linked to the cis-acting XmnI polymorphism (rs7482144), located at −158 bp upstream in the promoter region of γ-globin genes (5′HBG2) [23]. It is a single-nucleotide substitution (C>T) that results in enhanced transcriptional competence of the HBG2 promoter, having the ability to alter the regulatory architecture of the γ-globin promoter [15].

This variant may regulate gene expression by reducing binding of the repressor transcription factor BCL11A at its consensus site (TGACCA) around position −115 of the HBG2 and HBG1 promoters. This mechanism prevents γ globin gene silencing and increases HbF levels [21,22]. Functional studies show that the rs7482144 T allele promotes a more permissive chromatin state, which increases basal and stress-induced γ-globin transcription and makes it easier to recruit erythroid transcriptional activators [24].

Notably, rs7482144 primarily influences HBG2 expression, while BCL11A polymorphisms regulate both HBG1 and HBG2 [25]. There is no overlap between rs7482144 and the known binding motifs of BCL11A and ZBTB7A. While through the NuRD corepressor complex, BCL11A and ZBTB7A both suppress γ-globin expression, BCL11A binds a consensus motif close to −115 bp, while ZBTB7A binds closer to −200 bp upstream of the γ-globin transcription start site [26]. In addition, a region near the HBG2 promoter between −1445 and −1225 bp upstream has been found to vary across haplotypes and may further influence HbF levels.

For instance, in the Senegal/Benin haplotypes, modest HbF levels were associated with recombination breakpoints near −1500 bp upstream of HBG2; whereas higher HbF levels correlated with breakpoints downstream of positions −369 to −309 rs7482144 may impact the repressor ZBTB7A (binding site at −200 of the HBG promoter) or favor binding of activators such as GATA1, thereby contributing to HbF induction [3,13]. As a result, rather than directly interfering with repressor binding, the XmnI polymorphism has an indirect effect that may lower the effectiveness of repressor complex assembly or stabilize activator occupancy by slightly changing promoter topology or nucleosome positioning [27].

Comparative functional analyses reveal that HbF increases significantly more when BCL11A or ZBTB7A binding motifs are directly mutated than when rs7482144 is alone. This suggests that although the polymorphism plays a role, the lack of repressor sites has a greater impact [26,27]. Consequently, rs7482144 works both independently and in tandem with significant trans-acting repressors such as BCL11A and ZBTB7A to contribute to HbF induction at the molecular level by slightly increasing the permissiveness of the γ-globin promoter.

### 4.2. Polymorphisms Associated with Fetal Hemoglobin in Genome-Wide Association Studies (GWAS)

With the advent of NGS, GWAS has identified several genetic variants that modify HbF levels, as well as rarer loci with potential for future investigation. GWAS is a powerful tool for uncovering associations between common genomic variants; however, it is critical to note that the significant variants identified are not always causative but may instead be in linkage disequilibrium (LD) with the causal variant, which complicates interpretation [28].

To date, consistently associated genetic variants have been mapped to three major loci: the HBB cluster, BCL11A (chromosome 2p16), and the intergenic HBS1L-MYB region (HMIP, chromosome 6q23) [29]. BCL11A is the encoder of a zinc-finger transcriptional repressor that directly binds γ-globin gene promoters; it transcriptionally silences HbF in adult erythroid cells through the recruitment of chromatin-modifying corepressor complexes such as NuRD, which establishes a repressive epigenetic state at the HBG locus, causing fetal to adult hemoglobin switching [30,31]. On the other hand, the polymorphisms in the intergenic region of HBS1L-MYB reduce the transcription factor binding and alter enhancer elements that engage in long-range chromatin loopin, which regulates MYB transcription in erythroid progenitors, which causes delayed erithroid maturation and increases the persistence of HbF [32]. Together, polymorphisms in these regions, including rs7482144 (XmnI-HBG2), BCL11A variants, and HMIP polymorphisms, use direct γ-globin repression versus indirect enhancer-mediated modulation of erythroid differentiation to regulate HbF without modifying the globin structure and stability, and account for up to 50% of the heritable variation in HbF levels [28,33].

A recent systematic review (2007–2023) analyzed GWAS that investigated HbF-related variants in SCD and β-thalassemia [33]. The review identified 552 associations (*p*-value < 1 × 10^−5^), mapping to 133 genes (23 with overlapping variants) and 103 intergenic regions, strongly confirming the most implicated loci:

- Ten high-quality studies in SCD focusing on genetic regulators of HbF with well-defined phenotypes (HbF percentage or F-cell content).
- All SNPs were associated with changes in %HbF, and 17 also showed significant associations with %F-cells.

The strongest signals converged on BCL11A (23 SNPs) and HMIP (7 SNPs), with one SNP mapping to FHIT (chromosome 3) [34]. Rs7482144 (XmnI-HBG2) was specifically correlated with F-cell phenotype [35]. BCL11A variants showed the most consistent associations across cohorts, particularly rs766432 (replicated in eight cohorts) and rs10195871 (seven cohorts).

The most significant *p*-values were reported for BCL11A SNPs associated with %HbF: rs1427407 (*p* = 3.79 × 10^−53^; β = −0.3), rs6706648 (*p* = 4.96 × 10^−34^; β = −0.395), and rs7606173 (*p* = 8.50 × 10^−33^; β = −0.393) [28,36]. This is in line with their localization to an erythroid-specific enhancer, where HbF-increasing alleles de-repress γ-globin and induce HbF by reducing BCL11A expression [37].

Additionally, rs7606173 in BCL11A demonstrated the strongest association with %F-cells (*p* = 5.14 × 10^−16^; β = 1.5) [38].

SNPs in HMIP-2A, such as rs9399137 (*p* = 1.39 × 10^−24^; β = 0.45), further support the major regulatory contribution of the HBS1L-MYB intergenic region [39].

These findings underscore the pivotal role of BCL11A in regulating both %HbF and %F-cells across SCD cohorts. The directed disruption of BCL11A in erythroid cells reactivates HbF and reverses phenotypes of SCD in vivo, validating these regulatory variants as causal [28,37].

Table 3 summarizes the SNPs most consistently associated with HbF levels, as reported in the recent systematic GWAS review by Stephanou et al. [35]. These associations were defined by statistical significance (*p* < 1 × 10^−5^), replicated across multiple studies, confirmed in diverse populations, and supported by the magnitude of effect size reported in the included cohorts [33].

#### 4.2.1. Other Polymorphisms

Other polymorphisms, although described in fewer cohorts and with less consistent genomic significance, could be of interest in the modulation of HbF. These include variants in genes involved in oxidative stress response and erythroid transcriptional regulation:

- FHIT (chromosome 3)
- BACH2 and ALDH8A1 (chromosome 6)
- NFIX and ZBTB7A (chromosome 19) [40].

Recent research shows that NFIX acts as a repressor of γ-globin, and its silencing in erythroid cells would allow for increased HbF expression [41].

##### Rare Variants and Transcriptional Repressors

Recent research highlights the functional roles of these lesser-known repressors [41]:

- NFIX: Acts as a γ-globin repressor, where its silencing in erythroid cells would allow for increased HbF expression.
- KLF1: Loss of function mutations in this key erythroid transcription factor (observed in family studies of Hereditary Persistence of Fetal Hemoglobin, or HPFH) reduce the activation of the repressor BCL11A, thereby indirectly favoring γ-globin expression [42].

##### Chromatin Structure and Olfactory Receptor Genes

In addition to the above, Alghubayshi et al. [43] show that some SNPs in the olfactory receptor genes flanking the HBB locus have been associated with HbF levels in GWAS, suggesting that they may play a structural role in chromatin organization and modulation of the locus control region (LCR).

Within the β-globin cluster, *CBS* (CTCF binding sites), or CCCTC-binding factor, sites are critical:

- They are located at the boundaries of the β-globin cluster (3′HS1 and 5′HS5) and near the olfactory receptor genes (3′OR52A5 and 5′OR51B5).
- These sites anchor chromatin loops and determine the activation or silencing of globin genes.

Alteration of these CBSs modifies chromatin conformation and has revealed the presence of an HPFH enhancer located between 3′HS1 and 3′OR52A5, within the OR52A1 gene [44]. This enhancer promotes the activation of γ-globin genes (HBG1 and HBG2), thus contributing to HbF production.

All these findings highlight the importance of continuing to explore fewer common variants, and although they are not yet suitable for inclusion in predictive clinical models, they can serve as hypotheses in the development of personalized therapeutic strategies.

### 4.3. Gene Editing Strategy for Reactivating Fetal Hemoglobin

The analysis of classic haplotypes of the β-globin cluster and polymorphisms in regulatory regions such as intron 2 of BCL11A, the intergenic locus HBS1L-MYB, and the 7,482,144 Gamma 2 β-globin variant has provided insight into the regulation of γ-globin gene expression [45] and provided the molecular basis for the development of editing strategies to increase HbF levels as a protective factor against clinical complications in sickle cell disease [46].

Various genetic manipulation systems, such as zinc finger nucleases (ZFN), transcription activator-like effector nucleases (TALEN), and lentiviral gene transfer vectors, have contributed to early advances in β-globin gene correction and γ-globin activation [47].

However, this review focuses on three CRISPR-derived editing strategies:

- CRISPR Cas9
- Base editing
- Prime editing.

The following sections will explain in general terms how each strategy allows the γ-globin gene activation to boost HbF production.

#### 4.3.1. CRISPR-Cas9 Editing

This technique targets the inactivation of γ-globin repressors, such as BCL11A. By disrupting BCL11A, the repression of γ-globin genes is eliminated, allowing an increase in their expression and subsequently an increase in HbF levels [48].

The CRISPR-Cas9 system comprises two main components: the Cas9 protein and a guide RNA (sgRNA).

The sgRNA, as its name suggests, guides Cas9 to the region of DNA to be edited, in this case the BCL11A enhancer [49]. Upon reaching the editing site, the Cas9 protein produces a double-strand break (DSB) in the DNA, and the cell immediately attempts to repair this DSB using its dominant repair mechanism: non-homologous end joining (NHEJ) [50].

The incorporation of these insertions and deletions into the BCL11A enhancer alters its function, preventing it from being expressed correctly and thus allowing the activation of HbF [51].

#### 4.3.2. Base Editing

This strategy allows for the specific conversion of base pairs without inducing a double-strand break (DBS) and mimics the mutations that cause HPFH, which consist of point changes in the promoter of γ-globin genes (HBG1/HBG2), increasing transcription for HbF synthesis [52].

To the base edition, the system needs two key parts:

- A Cas9 nickase, which only binds to DNA or produces a single-strand break (SSB), rather than a DBS.
- A deaminase enzyme, capable of modifying specific nucleotides [53].

The Cas9-nickase complex is guided by sgRNA to the target region in the DNA, forming an R-loop that exposes a group of single-stranded nucleotides in the complementary strand. Subsequently, the deaminase enzyme acts on this strand, performing the desired base change [54].

By editing a specific base in the γ-globin promoter, the system generates much stronger binding sites for key transcriptional activators, such as GATA1, KLF1, and TAL1. This leads to the robust and improved transcription of the γ-globin gene and, consequently, to elevated and sustained levels of HbF [55].

#### 4.3.3. Prime Editing: A New Gene Editing Strategy

A recent study by Chalumeau et al. [56] demonstrated the use of prime editing in the reactivation of HbF, and, contrary to single-point editions, it allows multiple modifications within γ-globin promoters (HBG1 and HBG2), incorporating combinations of similar mutations to the HPFH (base conversions, insertions, or deletions) as well as those repressing the BCL11A transcription factor.

##### Molecular Mechanism for HbF Reactivation

The PE system utilizes a Cas9 nickase (Cas9n) fused to a Reverse Transcriptase (RT). The editing is guided by a prime editing guide RNA (pegRNA), which carries the desired edits within its RT template (RTT) [56,57].

The therapeutic strategy involves strategically combining mutations to achieve a synergistic effect:

- Activator Site Generation: Introducing single-base changes to create or strengthen binding sites (BSs) for key erythroid transcriptional activators, such as GATA1, KLF1, and TAL1, in the γ-globin promoter regions [58].
- Repressor Site Disruption: Simultaneously installing small deletions or indels (e.g., the Delta 5B or Delta 2B deletion of the BCL11A core motif) to disrupt repressor BSs, particularly BCL11A and Leukemia/Lymphoma-Related Factor (LRF) [58,59].

The difference with its predecessor CRISPR-Cas9 is the way the regulatory changes are introduced at the γ-globin promoters. While CRISPR-Cas9 induces DSBs at target sites, determining the final genomic outcome by repair through homology-directed repair (HDR) or non-homologous end joining (NHEJ), prime editing modifies the HBG1 and HBG2 promoter regions directed by pegRNA sequence rewriting (which enables simultaneous introduction of multiple HPFH-like mutations within the same region) without generating DSB. These approaches represent distinct molecular frameworks in HbF reactivation, differing primarily in how genetic changes are introduced and resolved at the DNA level [56,60].

##### Results and Efficacy in HSPCs

The resulting engineered promoters promote the sustained transcription of the γ-globin gene in erythrocytes derived from hematopoietic stem and progenitor cells (HSPCs). Critically, the functional analysis by Chalumeau et al. [56] demonstrated that erythroid clones carrying multiple edits exhibited a significantly higher γ-globin expression compared to cells carrying only single or individual mutations.

Specifically, optimization focused on enhancing the installation of complex edits (e.g., the GATA1/KLF1 BSs insertion combined with BCL11A deletion) by modifying the length and sequence of the RTT. The study found that the Prime Editor max system (using a Cas9 nuclease-PEmax construct) was particularly effective in HSPCs, reaching up to 7% of combined edits [57].

Although prime editing efficiency for complex edits can be lower than that of base editing for simple edits, the finding that multi-edited clones expressed higher text HBG levels than single base-edited colonies underscores the therapeutic potential of this comprehensive combinatorial approach to maximize HbF induction per corrected cell [56,57,58,59,60].

This technology is currently still in the experimental phase, with no real clinical applications yet realized.

The following Table 4 provides a comparison between prime editing technology and other CRISPR technologies studied for HbF modulation.

## 5. Conclusions

The modulation of fetal hemoglobin in sickle cell anemia represents a fundamental piece in the clinical manifestations of the pathology, understanding that at an increased level of HbF, a less severe clinical presentation the patient will have.

With the arrival of next-generation sequencing (NGS), genome-wide association studies (GWAS) have complemented and strengthened our knowledge of different modulators that could participate in the reactivation of HbF, highlighting BCL11A variations as one of the most potent and better-known regulators. In addition to the above, the presence of less common genetic modulators, such as chromatin regulatory elements and polymorphisms in transcription factors, could provide new perspectives for the development of treatments.

A study of the classic β-globin cluster and the presence of key polymorphisms in the modulation of HbF allows us to elucidate the phenotypical variation in SCD and has become a molecular base for the development of therapeutic interventions such as genic therapy, including mainly the CRISPR system and ranging from the deactivation of γ-globin repressors, such as BCL11A, all the way to the precise introduction of HPFH-like mutations in technologies such as base editing and prime editing, representing a revolutionary change in treatment and transforming the management perspective of the disease.

## Figures and Tables

**Table 1:** Molecular characterization of β-globin haplotypes.

Haplotype	rs3834466	rs7482144	rs10128556	rs968857	rs28440105
**ATP-I**	TT	C	C	C	C
**ATP-II**	TT	C	T	T	C
**ATP-III**	TT	C	C	T	C
**AI**	TT	T	T	T	C
**SEN**	T	T	T	T	C
**BEN**	T	C	C	T	C
**BAN**	T	C	C	C	C
**CAM**	T	C	C	T	A
**position GRCh38.p14 chr11:**	5270334	5254939	5242453	5239228	5248569
**Gene: consequence**	HBE1: 2KB upstream variant	HBG2: 2KB Upstream Variant	HBBP1: Intron variant	none	HBG1: Intron variant
**Variation Type:**	Insertion	Single-nucleotide variation	Single-nucleotide variation	Single-nucleotide variation	Single-nucleotide variation
**REF/(Frequency) ^†^**	T/(0.56722)	C/(0.73999)	C/(0.71468)	T/(0.57494)	A/(0.2699)
**ALT/(Frequency) ^†^**	TT/(0.43278)	T/(0.26001)	T/(0.28532)	A/(0.00000) C/(0.42506)	C/(0.7301) T/(0.0000)

Author’s elaboration based on data from [2,13,18]. NOTE = ^†^ Frequency of ref (reference) and alt (alternative) alleles based on the 1000 Genomes Project study.

**Table 2:** Sequencing technologies used in the identification of classical βs haplotypes.

Platform and Principle	Applicability	Advantages	Disadvantages	References
**1st-Generation** **Sanger** **Chain termination by dideoxy method** **Read length: 500–900 bp**	Exact polymorphism identification contrary to RFLP Useful for short fragment study, like those analyzed in the β-globin cluster Short turnaround time	Detects pathogenic genetic variants in the β-globin gene Low cost	Requires high technical skill from the lab technician Variable ability to detect large deletions Labor intensive and time consuming (~2 h)	[8,17]
**2nd-Generation** **Illumina** **Sequencing by synthesis method** **Read length: 2 × 150 to 2 × 300 bp**	Lower error rate in haplotype analysis Simultaneous screening for multiple loci to find modifier polymorphisms Whole genome study for patients with SCD Discovery of new SNPs that modify the disease phenotype	High performance Deep coverage in sequencing reads No PCR-amplification required Low error rate Long reads (>1000 bp) Enables the study of multiple variants	Requires high technical skill from the lab technician Labor intensive and requires expertise in equipment handling Generates large amounts of data that require a specialized software Requires bioinformatic experts for data analysis Expensive	[19,20,21]

**Table 3:** Key SNPs significantly associated with HbF levels in sickle cell disease.

Gene/Region	SNP (rsID)	Associated Allele	Location	Chromosome Location GRCh38	Variation Type	Effect on HbF	Association Statistical Data	Reference
**BCL11A**	rs1427407	G	Intron 2 Chr2 p16.1	2 p16: 60490908	SNV Single-Nucleotide Variation	HbF increase. F-cells increase. Explains 23% of fenotypical variation.	*p*-value: 3.79 × 10^−53^; β = −0.30	[33]
**BCL11A**	rs6706648	T	Intron 2 Chr2 p16.1	2p16: 60494905	SNV	HbF increase.	*p*-value: 4.96 × 10^−34^; β = −0.395	[28]
**BCL11A**	rs766432	A	Intron 2 Chr2 p16.1	60492835	SNV	HbF increase. Explains 5–11% of HbF variation.	*p*-value: <3.32 × 10^−13^	[33]
**BCL11A**	rs7606173	C	Intron 2 Chr2 p16.1	60498316	SNV	HbF increase.	*p*-value: 8.50 × 10^−33^; β = −0.393	[38]
**BCL11A**	Rs7606173	G	Intron 2 Chr2 p16.1	60498316	SNV	F-cells increase.	*p*-value: 5.14 × 10^−16^; β = 1.5	[38]
**HBS1L-MYB**	rs7776054	G	Intergenic (between HBS1L and MYB) Chr 6q23, HMIP	135097778	SNV	HbF increase.	*p*-value: 9.40 × 10^−164^; β = 0.51	[34]
**HBS1L-MYB**	rs9399137	T	Intergenic region HMIP-2A chr 6q23	135097880	SNV	HbF increase.	*p*-value: 1.39 × 10^−24^; β = 0.45	[39]
**HBS1L-MYB**	rs7775698	C	Intergenic region HMIP-2 chr 6q23	135097497	SNV	Associated with HbF.	*p*-value: 1.38 × 10^−23^ β = 0.44	[39]
**HBG2 (HBB)**	rs7482144 (XmnI-HBG2)	–158: T	HBG2 promoter chr11p15.4	5254939	SNV	HbF increase. Explains 4–8% of HbF variation. Associated with F-cells.	*p*-value: <1 × 10^−5^	[25]

**Table 4:** Genome editing strategies for fetal hemoglobin reactivation.

Characteristic	CRISPR/Cas9	Base Editing (BE)	Prime Editing (PE)
**Main mechanism**	Induces double-strand DNA breaks (DSBs), repaired by NHEJ or HDR.	Uses a Cas9 nickase/deactivated fused to a deaminase to catalyze base transitions without creating DSBs.	Cas9 nickase fused to a reverse transcriptase guided by pegRNA (PBS + RTT) to introduce substitutions, insertions, or deletions without DSBs.
**Possible mutations**	Insertions/deletions (InDels); gene knock-outs; larger sequence replacements via HDR.	Base transitions (C → T, A → G) limited to specific editing windows (puntual edition); does not allow insertions/deletions.	Precise substitutions, insertions, deletions; simultaneous introduction of multiple HPFH-like mutations in HBG1/2 promoters.
**Example in HbF reactivation**	Disruption of BCL11A repressor sites; used in clinical exa-cel therapy.	Introduction of single HPFH-like point mutations in γ-globin promoters compatible with editing windows.	Insertion of multiple HPFH/HPFH-like mutations into HBG1/2 promoters; erythroid clones carrying combined edits showed increased γ-globin expression.
**Editing efficiency**	High in many contexts; variable mosaicism depending on repair pathway; clinically relevant efficiencies achieved.	High for compatible base transitions; dependent on deaminase and locus context.	Up to ~50% precise edits in hematopoietic cell lines; variable in patient HSPCs; ~34% achieved with optimized pegRNAs/epegRNAs.
**Specificity Off-target effects**	Can generate significant off-target cleavage; DSBs may cause large rearrangements.	Lower off-target activity compared to nucleases; risk of unintended bystander edits within editing window.	Generally specific with minimal off-targets reported; however, InDels and large deletions (e.g., 4.9 kb between HBG1–HBG2) also observed.
**Safety Technical limitations**	Risk of chromosomal translocations, large deletions, p53 activation, and genotoxicity.	Restricted to four types of base transitions; limited applicability for insertions/deletions.	Variable efficiency, scaffold incorporation, partial edits; generation of InDels and long deletions; optimization of pegRNAs required.
**Clinical status**	Approved clinical trials (exa-cel, BCL11A knockout) for β-hemoglobinopathies.	Preclinical use; not yet clinically approved for HbF reactivation.	Experimental; proof-of-concept in HSPCs; no clinical application yet.

## Data Availability

No new data were created or analyzed in this study. Data sharing is not applicable to this article.

## Abbreviations

The following abbreviations are used in this manuscript:

BEs	Base Editors
BS	Binding Site
CBS	CTCF binding site
CTCF	CCCTC-binding factor
DBS	Double-strand break
epegRNA	Engineered pegRNA
GWAS	Genome-wide association studies
HbF	Fetal hemoglobin
HbS	Sickle hemoglobin
HR/HDR	Homologous recombination/homology-directed repair
HSPCs	Hematopoietic stem and progenitor cells
HPFH	Hereditary persistence of fetal hemoglobin
InDels	Insertions and deletions
LD	Linkage disequilibrium
NGS	Next-generation sequencing technologies
NHEJ	Non-homologous end joining
PE	Prime editing
PBS	Primer binding site
pegRNA	Prime Editing Guide RNA
RFLP	Restriction fragment length polymorphism
RNA	Ribonucleic acid
RTT	Reverse transcriptase template
SCD	Sickle cell disease
sgRNA	Guide RNA
SNP	Single-nucleotide polymorphism
SSB	Single-strand break
TALEN	Transcription activator-like effector nucleases
WHO	World Health Organization
ZFN	Zinc finger nucleases
